# Current Trends in Management of Nonsyndromic Unilateral Coronal Craniosynostosis: A Cross-sectional Survey

**DOI:** 10.1097/GOX.0000000000002229

**Published:** 2019-05-16

**Authors:** Christophe Moderie, Alexander Govshievich, Frank Papay, Jeffrey Fearon, Arun Gosain, Gaby Doumit

**Affiliations:** From the * Faculty of Medicine, University of Montreal, Montreal, Quebec; † Department of Plastic Surgery, University of Montreal, Montreal, Quebec; ‡ Department of Plastic Surgery and Dermatology, Cleveland Clinic, Cleveland, Ohio; § The Craniofacial Center, Medical City Dallas Hospital, Dallas, Tex.; ¶ Division of Plastic Surgery, Lurie Children’s Hospital, Chicago, Ill.

## Abstract

**Background:**

Although the natural history of nonsyndromic unilateral coronal craniosynostosis has been extensively described, optimal management remains controversial due to lack of Level 1 evidence. This study aims to assess the current state of practice among craniofacial surgeons.

**Methods:**

Ninety-four craniofacial surgeons were approached to complete a survey consisting of 15 questions. Data were collected assessing surgeons’ primary surgical indication, timing of intervention, preoperative imaging, and choice of technique for patients presenting with nonsyndromic unilateral coronal craniosynostosis. Choice of technique and timing of intervention in case of recurrence were also investigated.

**Results:**

After 5 mailings, the response rate was 61%. The combination of both appearance and raised intracranial pressure was the primary indication for treatment for 73.2% of surgeons. Preoperative CT scan of the skull was “always” performed by 70.1% of respondents. Open surgical management was most commonly performed at 8–10 months of age (38.6%). Bilateral frontal craniectomy with remodeling of the supraorbital bandeau and frontal bone was the most common choice of procedure (84.2%). In case of mild to moderate and moderate to severe recurrences at 1 year of age, 89.5% and 47.4% of surgeons opted for conservative management, respectively. Optimal timing for repeat cranioplasty was after 4 years of age (65.5%). Overall, 43.4% quoted lack of evidence as the greatest obstacle to clinical decision-making when dealing with unilateral synostosis.

**Conclusion:**

This survey exposes the lack of consensus and the disparity of opinion among craniofacial surgeons regarding the management of nonsyndromic coronal synostosis, particularly in the setting of recurrence.

## BACKGROUND

Unilateral coronal craniosynostosis (UCS) and metopic synostosis are the most common forms of craniosynostosis following sagittal synostosis. UCS occurs in 1 of 10,000 live births.^1–3^ Premature fusion of the coronal suture combined with the rapidly expanding infant brain results in the characteristic morphology of anterior plagiocephaly. These findings, which range from mild to severe, include ipsilateral frontal and posterior flattening and superoposterior displacement of the supraorbital ridge with compensatory contralateral frontal bossing. Radiologically, an undescended greater wing of the sphenoid on the affected side results in the characteristic Harlequin deformity.

The primary goals of treatment in UCS are restoration of normal skeletal anatomy in the fronto-orbital region, minimization of severity of future facial scoliosis, and possible prevention of high intracranial pressure side effects on the brain. General surgical principles include advancement of the ipsilateral superior and lateral orbital rims, reduction of ipsilateral orbital height, and correction of the frontal deformity. Despite our understanding of the anatomy, natural history, and pathophysiology of this condition, optimal management remains controversial. Due to the relative scarcity of UCS, randomized controlled trials are difficult to execute, as involvement of multiple centers would be required. Most available literature regarding the surgical treatment of this pathology comprises retrospective studies and case series. As a result, there is a wide disparity of opinions regarding the best practice for treatment of nonsyndromic coronal synostosis.

The aim of the current study was to evaluate current management of this condition among experienced craniofacial surgeons by using a cross-sectional survey. A better understanding of actual trends and the establishment of a consensus opinion might lead to an improvement of the medical care.

## METHODS

Ninety-four craniofacial surgeons were approached to complete a cross-sectional survey. At least 5 years of a pediatric-oriented practice was required to participate in the study. All queried surgeons were members of the International Society of Craniofacial Surgery. Up to 5 reminders were sent to nonresponders. The internet-based survey consisted of 15 multiple-choice questions assessing surgeons’ primary indication for surgery, preference of timing, and choice of operative procedures for patients presenting with nonsyndromic UCS (see Table 1). Further, questions regarding timing and management of recurrence were included. Preoperative and postoperative management were also investigated.

**Table 1.**
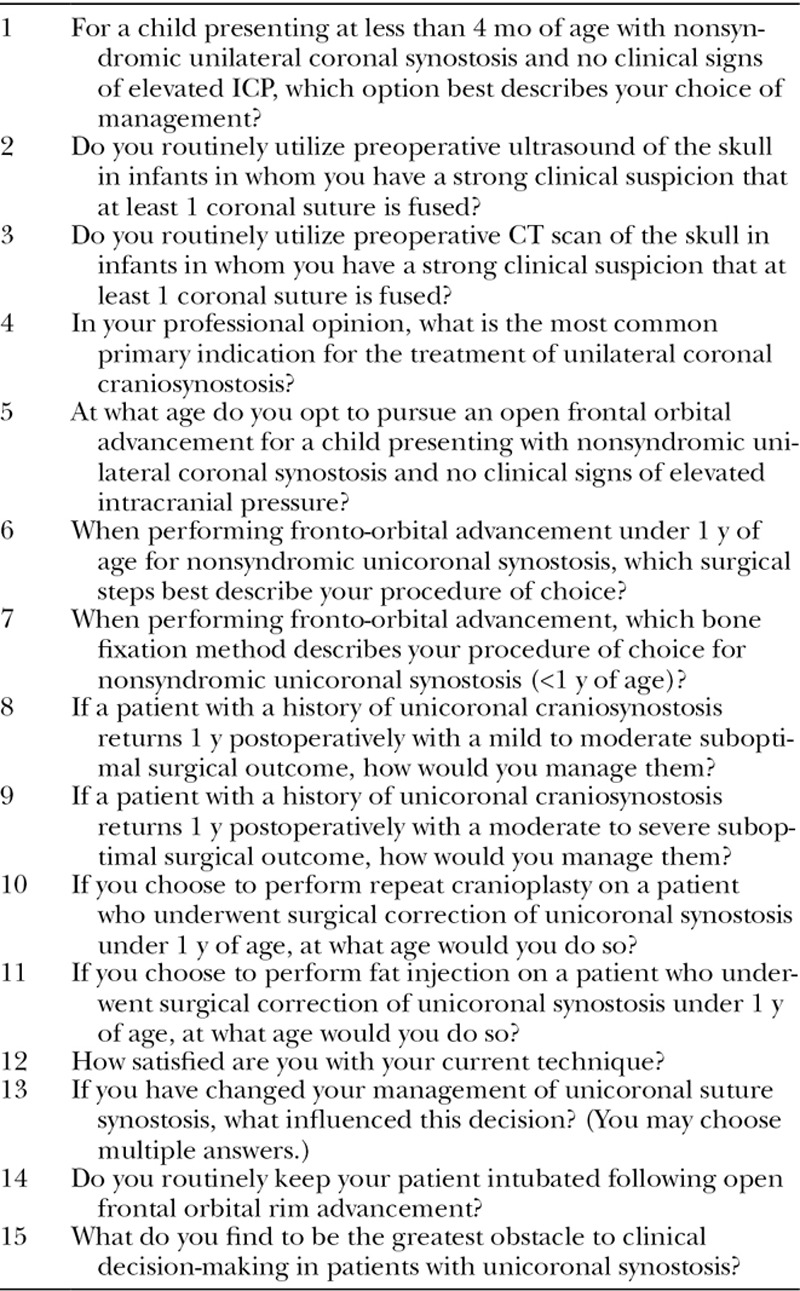
Survey Questions

## STATISTICAL ANALYSIS

Direct question assay and cross-referencing were used to analyze obtained data. The associations between the variables were compared using chi-square tests. Statistical significance was determined at *P* < 0.05. All statistical calculations were carried out using IBM SPSS Statistics for Windows (Version 22.0. Armonk, N.Y.). Except where otherwise specified, data are expressed as mean ± SD.

## RESULTS

### Preoperative Evaluation Techniques

A response rate of 61% (57/94) was obtained after 5 mailings. Concerning the preoperative assessment, our survey demonstrated that in the evaluation of patients who are felt to have UCS based on clinical presentation, 70.1% (n = 40) of polled surgeons always obtained CT scans, 19.3% (n = 11) only used CT scan when the physical examination was inconclusive, and 3.5% (n = 2) never used CT scan. Ultrasonography (US) of the skull was systematically used by only 2 (3.5%) surgeons. It was used in those for whom physical examination did not provide a clear diagnosis by 5 (8.8%) other surgeons. The remaining 50 surgeons (87.5%) almost never used ultrasound in the preoperative evaluation of UCS.

### Choice of Management

The primary indication for treatment of craniosynostosis was both appearance and possible raised pressure for the majority of the surgeons (73.2%). Some relied only on craniofacial scoliosis alone (ie, appearance; 21.4%) whereas a minority (5.4%) reported neurocognitive disability or delay as their primary indication for treatment.

### Surgical Procedures

When performing fronto-orbital advancement, bilateral frontal craniectomy with remodeling of the supraorbital bandeau and frontal bone (84.2%) was more often used than unilateral frontal craniectomy with remodeling of the supraorbital bandeau and frontal bone (3.5%). To perform an orbital rim advancement, the detachment of the orbital rim was more often reported to be done at the frontal zygomatic (24.6%) suture. Some preferred to proceed by detaching the lateral orbital rim inferior to the frontal zygomatic suture (16.1%). The latter tended to be more satisfied with their technique than the former (*P* = 0.79). A minority reported preferring supraorbital rim advancement with no detachment of the contralateral supraorbital bandeau (7%, n = 4). An interposition bone graft and inlay bone strut to orbital roof was performed by some (17.5%, n = 10), while a few others used the parietal bone to substitute the frontal bone and remodeled the supraorbital bandeau (10.5%, n = 6), and a few used the parietal bone to reconstruct both the supraorbital bandeau and the forehead (5.3%, n = 3). One respondent reported remodeling the frontal bone for the supraorbital bandeau and use the parietal bone for the forehead.

Concerning the bone fixation method, less disparity was noted. Indeed, the vast majority used resorbable plates (91.2%) compared to titanium plates (1.8%). Similarly, resorbable sutures were used more often than stainless steel sutures (43.9% versus 12.3%, respectively). Strip craniectomy of the fused coronal suture followed by helmet (3.5%, n = 2) or distraction device/springs (5.3%, n = 3) were the least commonly used methods.

Keeping the patient intubated postoperation is rare, as revealed by the fact that 91.2% of the respondents almost never do it. The remaining 8.8% only keep their patients intubated if they require extensive blood transfusion, prolonged surgical procedure, or suffered an intraoperative complication.

### Timing of Surgery

In the absence of clinical signs of elevated ICP for a child of less of 4 months of age, 61.7% of the respondents would delay the operative intervention until after 6 months of age. There was no consensus on the specific range of age to pursue the intervention although 96.5% would proceed to an open frontal orbital advancement between ages 5 and 13 months. The most popular timeframe to intervene was 8–10 months (38.6%) followed by the 5- to 7-month timeframe (31.6%). The satisfaction of the surgeons in regard to the outcome of the management did not differ according to the timing of operation. A total of 61.1% and 63.6% of surgeons were very satisfied with their technique when operating at 5–7 and 8–10 months of age, respectively (*P* = 0.63).

### Follow-up

In case of a mild to moderate (Whitaker 2–3) clinical recurrence 1 year postoperatively, a vast majority of surgeons (89.5%) agreed to follow conservatively and reevaluate the patient. Four surgeons (7.0%) would plan to perform fat injection to the forehead at a later date. Two surgeons (3.5%) stated that they would repeat the cranioplasty procedure. One surgeon would reoperate with synthetic onlay or bone paste, and another respondent opted to wait until around 9–10 years of age and then modify the forehead surgically.

There was no consensus on the situation of moderate to severe (Whitaker 3–4) clinical recurrence 1 year postoperative. Conservative management and reevaluation were the most popular approaches (47.4%) followed by reoperation, either by performing cranioplasty (29.8%) or augmentation with synthetic onlay or bone paste (1.8%). When choosing to perform repeat cranioplasty, the age of reoperation varied greatly across surgeons, although the majority (65.5%) of surgeons would reoperate at the age of 4 or older. Helmet treatment was used by 1 surgeon. The remaining surgeons (5.3%) opted for fat injection to the forehead at a later date. The majority of surgeons (92.5%) preferred to perform the fat injection at age 5 years or greater.

## DISCUSSION

Various techniques have been developed to address unilateral nonsyndromic craniosynostosis, but the lack of high level evidence supporting the superiority of a technique over another has led to discrepancies in practice. Previous studies have shown the heterogeneity of views among surgeons concerning both sagittal and metopic craniosynostosis.^4–6^ Furthermore, it was found that clinical practice differed from recommendations in the literature regarding the management of sagittal craniosynostosis.^4^ The current approach of craniofacial surgeons toward nonsyndromic coronal craniosynostosis remains similarly controversial. Hence, the aim of this study was to shed some light on the current trends in practice regarding the preoperative evaluation techniques, choice of management, surgical procedures, timing of surgery, and follow-up for nonsyndromic coronal craniosynostosis.

### Preoperative Evaluation Techniques

Imaging techniques are a precious tool to evaluate patients and to plan any required intervention. Although physical examination alone has a 98% accuracy to diagnose nonsyndromic craniosynostosis,^7^ the CT scan is still frequently used among clinicians in their preoperative workups.^4–6^ These scans provide the highest diagnostic accuracy, may demonstrate signs of increased intracranial pressure, and can aid in surgical planning. In our survey, CT scan was routinely used by 70.2% of the surgeons. Nonetheless, 19.3% of respondents reported relying primarily on physical examination, using CT scan only when the clinical diagnosis was inconclusive.

A nonradiating imaging modality alternative to preoperative CT is ultrasound. In our survey, this method was unpopular among surgeons, and was never used by 87.7% of the respondents, which is similar to what has been previously reported in the literature.^5^ Perhaps more emphasis should be put toward implementing this modality in the clinical settings, considering the numerous studies that showed its accuracy to detect or rule out craniosynostosis.^8–10^ Alizadeh et al. found a sensitivity of 96.9% and a specificity of 100% when using US in 44 children aged <1 year with a diagnosis of synostosis.^11^ However, a major downside for the use of ultrasound is that it provides no information regarding surgical planning or virtual surgical planning when chosen. In addition, experience of radiologists in interpreting US varies, and radiologists who are not familiar with reading suture architecture on US may be confused when sutural architecture is obscured within segments of the suture being examined.

### Choice of Management

Left untreated, isolated craniosynostosis has 2 main consequences: (1) it causes craniofacial deformity which can lead to cosmetic defects and psychosocial sequelae^12^ and (2) brain growth in a limited space can result in increased ICP in up to 24% of the patients.^13^ Although it seems intuitive that the absence of intervention could be linked to mental disability, this association has not been clearly established. Some have reported that treated patients are more prone to develop cognitive disabilities than the general population.^14,15^ In line with what was reported in the literature, craniofacial surgeons identified both appearance and possible raised ICP as the (73%) primary indication for treatment and some reported appearance only to be their main indication for surgery (23%). Overall, appearance played a role in 96% of decisions to operate.

### Surgical Procedures

**Fig. 1.**
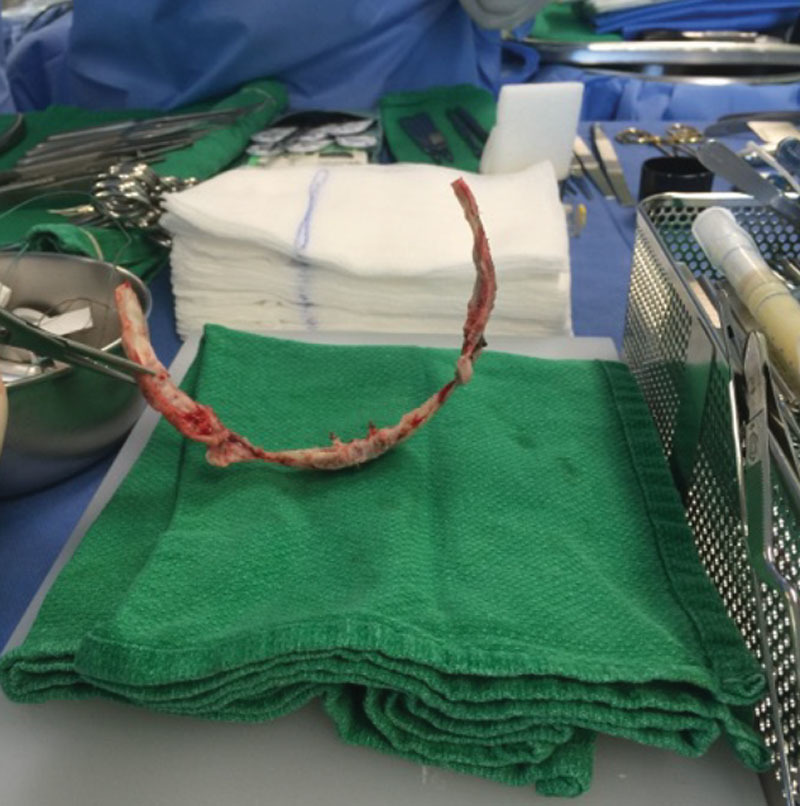
Intracranial migration of titanium plate and screws.

Unilateral coronal synostosis can be managed by cranial vault reconstruction or by minimally invasive procedures. Cranial vault reconstruction has the advantage to allow immediate reshaping of the head, and immediate alleviation of increased intracranial pressure when present. The overcorrection during the surgery could, furthermore, prevent relapse.^16^ The cranial vault is reconstructed by achieving a fronto-orbital advancement. To do so, bilateral frontal craniectomy with remodeling of the supraorbital bandeau and frontal bone was the most common approach (83.9%). The orbital rim was more often advance by detaching it at the frontal zygomatic suture. Fixation was typically done by using resorbable plates and sutures to maintain an overcorrected shape. Resorbable plates are particularly important in the pediatric population to prevent impeding bony growth. Nonresorbable material such as Titanium has been known to migrate intracranially as the child grows (Fig. 1).

Craniosynostosis can also be managed by minimally invasive procedures, usually at younger age when the skull is malleable and the diploic space is small, allowing for minimal blood loss.^17^ Minimally invasive and endoscopically assisted strip craniectomy was used by 15% of the respondents, whereas no surgeons reported using endoscopic-assisted frontal orbital advancement.^17,18^ Despite reducing the intraoperative visual field and requiring postoperative helmet molding therapy, the minimally invasive and endoscopically assisted approach offers considerable perioperative advantages.^4^ Of note, minimally invasive strip craniectomies have lower morbidity rates, operative duration, and transfusion rates than cranial vault reconstructive procedures.^17,18^ Reduced scarring and minimized parental anxiety are among other potential benefits of this approach.^4^ Longer term assessments will be necessary to determine the ideal treatment approach.

Another technique that can be used is the spring-assisted coronal strip craniectomy, which consists of inserting spring distractors within the space formed by the removal of a strip of the coronal suture.^19^ Lauritzen et al. reported improvement of the condition of patients 6 months after undergoing this procedure.^20^ In line with previous studies, no surgeons in the present sample opted for spring-assisted coronal strip craniectomy for a child of less than 4 months of age.^5^ The need to perform a second procedure and the unpredictability of the springs is among possible explanations for the lack of adoption of this technique.^4^

### Timing of Surgery

As for the timing of the surgical management, it is usually recommended to intervene before 1 year of age, which is done by almost all of the surveyed surgeons.^21^ The majority of the surgeons chose to operate between the ages of 8–10 months of age (38.6%). This contrasts with previous surveys namely on the management of sagittal synostosis where respondents opted to intervene earlier with 45% choosing to operate between 5 and 7 months of age.^4,6^ This could be linked to the fact that secondary surgical corrections are less frequent when operating at a later age because cranial bones are firmer and can better support the cranial vault remodeling.^21,22^ Older children also have a greater total blood volume.^16^ Nonetheless, when questioned about their management of a child of less than 4 months of age, 23.3% would proceed right away to an open surgical approach and 15% by proceeding to an extended strip craniectomy. Earlier intervention benefits from thinner bones and rapidly growing brain and could, therefore, minimize subsequent skull deformities and compensatory facial changes induced by brain growth.^23^

### Follow-up

Routine follow-up is indicated until skeletal maturity is reached.^13^ In case of contour irregularities, cosmetic abnormality, or persistence of bone defects, secondary cranioplasty might be indicated.^24^ When physicians were queried about their management of a suboptimal surgical outcome, their answers varied according to the severity of the clinical recurrence. In case of mild to moderate recurrence, conservative management was by far the most popular approach (89.5%). Regarding moderate to severe recurrence, little consensus exists. Many surgeons (46%) would follow conservatively and some (32%) would reoperate, which falls in the range of what has previously been reported (6%–36%).^25^

The helmet and the fat injection were the least commonly reported treatments. Although this technique can be repeated, is minimally invasive, is reported to have few complication and a fast recovery,^26^ some surgeons express concern about the long-term symmetry and contour irregularities that may result from this option.

### Satisfaction

In the present survey, 60.7% of surgeons reported a very high level of satisfaction in regard to their approach to UCS. The remaining (39.3%) were ‘‘somewhat dissatisfied’’ with their current technique and would change it if there more clear evidence-based medicine (EBM). This level of dissatisfaction is higher than the 22.2% of satisfaction found in our previous survey assessing management of sagittal craniosynostosis.^4^ When queried about the challenges in decision-making, lack of evidence to support alternate means was identified by 43.4% of the respondents. Accordingly, 35.2% of the surgeons highlighted the lack of consensus in the surgical society. Nonetheless, to date, only 24.5% of the surgeons that changed their management of UCS based this decision on EBM. Most respondents (48.2%) relied on their personal follow-up to change their management of UCS. Expert opinion also had a greater influence than EBM in changing techniques, whether it was a respected colleague providing advice during informal discussion or a senior (21.1%), well-respected craniofacial surgeon who provided personal evidence during a society meeting (15.8%). Interestingly, none of the respondents believed that they lacked an appropriate source of training by which to alter their technique for surgical correction of UCS. This highlights the fact that limitations regarding the management of UCS most probably result from insufficiency of evidence rather than technical limitations on the part of the treating surgeon.

The limitations of this study included lack of objective verification of reported management of craniosynostosis, the use of an online survey, its cross-sectional nature, and reliance on surgeons’ recall. The response rate was not considered a limitation. Although no scientifically proven minimally acceptable value has been established, our response rate of 61% stands above the threshold of acceptability and has face validity as a measure of survey quality.^27^ The quality is also upheld by the similarity of the survey population, consisting of pediatric-oriented craniofacial surgeons with at least 5 years of a practice. The similarity between respondents and nonrespondents minimizes the response rate bias and impacts the validity and representativeness of a survey to a lesser extent. As such, the authors believe these data are reflective of the scope of current management.

In summary, this survey exposes the lack of consensus and the disparity of opinion among craniofacial surgeons regarding management of nonsyndromic coronal synostosis, in particular with regard to timing of primary surgery and management of recurrence. A significant portion of surgeons are dissatisfied with current surgical technique and are open to change were the appropriate EBM available. Currently, changes in practice are largely influenced by personal follow-up and expert opinion. The lack of EBM and lack of consensus within the surgical community are considered to be the greatest obstacles to clinical decision-making in the management of UCS.
